# Nonlinear Dynamics of MEG and EMG: Stability and Similarity Analysis

**DOI:** 10.3390/brainsci15070681

**Published:** 2025-06-25

**Authors:** Armin Hakkak Moghadam Torbati, Christian Georgiev, Daria Digileva, Nicolas Yanguma Muñoz, Pierre Cabaraux, Narges Davoudi, Harri Piitulainen, Veikko Jousmäki, Mathieu Bourguignon

**Affiliations:** 1 Laboratory of Functional Anatomy, Faculty of Human Motor Sciences, Universite Libre de Bruxelles (ULB), 1070 Brussels, Belgium; christian.georgiev@ulb.be (C.G.); daria.digileva@ulb.be (D.D.); nicolas.yanguma.munoz@ulb.be (N.Y.M.); pierre.cabaraux@ulb.be (P.C.); 2 Laboratoire de Neuroanatomie et Neuroimagerie Translationnelles, UNI—ULB Neuroscience Institute, ULB, 1070 Brussels, Belgium; 3 Department of Neurology, Neurorehabilitation Ward, Hopital Universitaire de Bruxelles (HUB), ULB, 1070 Brussels, Belgium; 4 Department of Physics “Ettore Pancini”, University of Naples Federico II, 80126 Naples, Italy; narges.davoudi@yahoo.com; 5 Faculty of Sport and Health Sciences, University of Jyväskylä, 40014 Jyväskylä, Finland; harri.t.piitulainen@jyu.fi; 6 Department of Neuroscience and Biomedical Engineering, Aalto University School of Science, 02150 Espoo, Finland; veikko.jousmaki@aalto.fi; 7 Aalto NeuroImaging, Aalto University School of Science, 02150 Espoo, Finland; 8 WEL Research Institute Avenue Pasteur 6, 1300 Wavre, Belgium

**Keywords:** beta power, nonlinear dynamics, Lyapunov exponents, fractal dimension, correlation dimensions

## Abstract

**Background:** Sensorimotor beta oscillations are critical for motor control and become synchronized with muscle activity during sustained contractions, forming corticomuscular coherence (CMC). Although beta activity manifests in transient bursts, suggesting nonlinear behavior, most studies rely on linear analyses, leaving the underlying dynamic structure of brain–muscle interactions underexplored. **Objectives:** To investigate the nonlinear dynamics underlying beta oscillations during isometric contraction. **Methods:** MEG and EMG were recorded from 17 right-handed healthy adults performing a 10 min isometric pinch task. Lyapunov exponent (LE), fractal dimension (FD), and correlation dimension (CD) were computed in 10 s windows to assess temporal stability. Signal similarity was assessed using Pearson correlation of amplitude envelopes and the nonlinear features. Burstiness was estimated using the coefficient of variation (CV) of the beta envelope to examine how transient fluctuations in signal amplitude relate to underlying nonlinear dynamics. Phase-randomized surrogate signals were used to validate the nonlinearity of the original data. **Results:** In contrast to FD, LE and CD revealed consistent, structured dynamics over time and significantly differed from surrogate signals, indicating sensitivity to non-random patterns. Both MEG and EMG signals demonstrated temporal stability in nonlinear features. However, MEG–EMG similarity was captured only by linear envelope correlation, not by nonlinear features. CD was strongly associated with beta burstiness in MEG, suggesting it reflects information similar to that captured by the amplitude envelope. In contrast, LE showed a weaker, inverse relationship, and FD was not significantly associated with burstiness. **Conclusions:** Nonlinear features capture intrinsic, stable dynamics in cortical and muscular beta activity, but do not reflect cross-modal similarity, highlighting a distinction from conventional linear analyses.

## 1. Introduction

The role of beta oscillations in motor control is widely documented [1]. Beta oscillations decrease in power just before and during voluntary movements, reflecting cortical activation, and subsequently rebound to pre-movement levels as the motor system returns to a state of stability [2,3]. During steady sustained muscle contraction, beta oscillations are coupled with muscle activity, a phenomenon known as corticomuscular coherence (CMC) [4]. CMC has been mainly investigated through the use of linear methods such as Fourier-based coherence analysis. However, beta oscillations are known to present with transient bursts of few cycles separated by more silent periods [5,6,7], a behavior characteristic of complex, often nonlinear systems [8]. Despite this, the nonlinear temporal structure of the brain–muscle interactions during motor tasks remains poorly understood, representing a significant research gap. To address this limitation, the present study applies nonlinear dynamic analysis to cortical and muscular beta-band activity, focusing on the temporal evolution of dynamic complexity during an isometric contraction task. To characterize this complexity, we introduce several nonlinear measures that have proven effective in broader neurophysiological research.

Specifically, one approach to investigating the complexity and stability of beta oscillations consists of studying Lyapunov exponent (LE), correlation dimension (CD), and fractal dimension (FD) from the reconstructed phase space of electroencephalographic (EEG) or magnetoencephalographic (MEG) signals [9,10]. These measures have been widely used to assess cognitive and neural states. For example, LE has been shown to decline with age [11] and differentiate between levels of consciousness under anesthesia [12]. It has also been proposed as a biomarker in neurodegenerative and psychiatric conditions such as Alzheimer’s [13], Parkinson’s [14], and schizophrenia [15]. Similarly, FD has been used to capture the chaotic, self-similar nature of EEG signals [16,17], distinguish clinical populations such as stroke and epilepsy patients [18,19,20], and assess task-related complexity. CD, in turn, has revealed associations with intelligence [21], cognitive states [22], hemispheric engagement [23], and anesthesia depth [24], and has proven useful in applications such as motor imagery classification [25] and expertise assessment [26]. Despite their proven usefulness in other neural contexts, the utility of these nonlinear measures has not yet been tested to assess corticomuscular dynamics during steady isometric motor tasks.

Furthermore, the existence of CMC implies that there is some degree of similarity between both EMG and the brain signals (MEG and EEG) during steady muscle contraction, suggesting that EMG signals could be considered as proxy brain signals [27,28]. However, none of these studies examined the dynamics of these signals or their relationship to determine whether they share or follow similar dynamic patterns over time. More interestingly, many studies showed that neural synchronization between the brain and muscles occurs in short bursts of frequency-specific connectivity, even during sustained motor tasks. So, averaging over time windows may not be the most effective analysis approach [29]. Indeed, it remains unclear whether MEG and EMG signals exhibit temporally stable or fluctuating nonlinear dynamics throughout isometric motor execution. If the dynamic properties of the signals vary significantly over time, time-averaged approaches may obscure important transient features. Conversely, if they remain stable, then traditional averaging techniques may still be appropriate.

This study was designed to address three primary research questions:

1. Do LE, CD, and FD reliably reflect a nonlinear structure in MEG and EMG beta oscillations during isometric contraction?

2. Do beta oscillations in MEG and EMG signals exhibit stable nonlinear dynamic properties (LE, CD, FD) over time during isometric contraction?

3. Do these two signals share similar patterns of nonlinear dynamics during the task?

4. How do these nonlinear features compare with conventional analyses such as beta-band envelope correlation and envelope-based burstiness assessment?

We hypothesized that LE, CD, and FD, would serve as valid indicators of a nonlinear structure in MEG and EMG beta-band signals during steady isometric contraction, based on their established utility in prior neurophysiological studies. Additionally, we expected beta-band MEG and EMG signals would display similar nonlinear dynamic patterns over time and that these patterns would remain relatively stable during the contraction period. This hypothesis is based on the established presence of CMC, which implies functional coupling and synchrony between brain and muscle activity during steady contractions [27,28]. Moreover, previous findings of transient beta bursts suggest that the underlying dynamics, while complex, may be coordinated across these signals, reflecting shared nonlinear mechanisms [29].

In the current study, we assessed the time evolution of LE, CD, and FD based on the reconstructed phase space of beta-band MEG and EMG signals while healthy humans held an isometric contraction. We then assessed the stability of these parameters over time and compared them between MEG and EMG. Finally, the results were compared with those of a linear method assessing the similarity of MEG and EMG envelopes in the beta band.

## 2. Materials and Methods

This study is a reanalysis of previously published data [4].

### 2.1. Participants

A total of 17 healthy adult volunteers (10 males, 7 females; mean age ± SD: 34 ± 7 years, range: 20–47 years) with no history of neuropsychiatric disorders or movement impairments took part in the study. All participants were right-handed, with an average handedness score of 90 ± 12 (range: 65–100) on the Edinburgh Handedness Inventory scale (−100 to 100) [30].

Participants were recruited via university-wide advertisements and by word of mouth. Inclusion criteria were (1) age between 20 and 50 years, (2) right-handedness, and (3) no self-reported neurological or musculoskeletal conditions. Exclusion criteria included current or prior upper limb injuries, use of medication that could influence neuromuscular performance, and regular involvement in activities involving fine motor skills such as musicians, professional typists, and athletes.

### 2.2. Experimental Protocol

**Figure 1:**
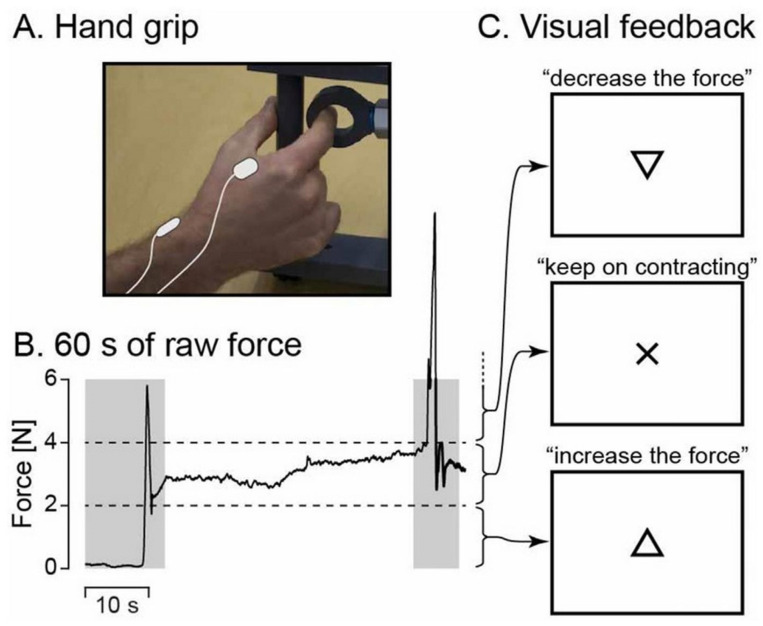
Experimental setup. Illustration of the isometric contraction task. A steady contraction is maintained on a custom-made handgrip with the right thumb and the index finger. Reproduced from [4].

Figure 1 depicts the experimental setup. During MEG recordings, participants were seated with their left hand resting on their thighs and their right hand positioned on a table in front of them. If necessary, vision was optically corrected using nonmagnetic goggles. Participants were instructed to maintain a steady isometric pinch grip of 2–4 N using a custom-made handgrip (connected to a rigid load cell with stiffness of 15.4 N/mm; Model 1004, Vishay Precision Group, Malvern, PA, USA) with their right thumb and index finger (Figure 1A), and to fixate at a black cross displayed on the center of a screen placed 1 m in front of them. If the applied force exceeded the prescribed limits, a triangle (pointing upward or downward) appeared above the black cross, indicating the direction in which the force needed to be adjusted. The triangle disappeared as soon as the force was correctly brought back within the designated range (see Figure 1B,C). After an ~1 min practice session, two 5 min blocks were recorded, with a minimum of 2 min rest between the blocks. Each block began with approximately 10 s of rest before participants were instructed to initiate the contraction task. Additionally, a 5 min task-free block was recorded. Notably, the chosen force range (2–4 N) allowed participants to sustain the contraction with minimal need for corrective feedback.

To minimize the effects of muscular fatigue, the contraction force level was deliberately set to a low intensity (2–4 N), which was confirmed in pilot testing to be sustainable for up to 5 min without inducing fatigue. Participants were asked to verbally confirm absence of fatigue before beginning each block, and a minimum rest period of 2 min was provided between blocks. The experimental room environment was controlled to minimize external stimuli, and temperature was maintained at approximately 22 °C, with dim lighting and no external noise or distractions.

### 2.3. Measurement

**MEG.** MEG recordings were conducted in a magnetically shielded room (Imedco AG, Hägendorf, Switzerland) at the MEG Core of Aalto Neuroimaging, Aalto University. Data were acquired using a 306-channel whole scalp neuromagnetometer (Elekta Neuromag™, Elekta Oy, Helsinki, Finland) with a recording passband of 0.1–330 Hz and a sampling rate of 1 kHz. Participants’ head position within the MEG helmet was continuously tracked by passing current through four head-tracking coils placed on the scalp. The positions of these coils, along with at least 200 head surface points (covering the scalp and nose), were recorded relative to anatomical fiducials using an electromagnetic tracker (Fastrack, Polhemus, Colchester, VT, USA).

**EMG**. Surface EMG was recorded from the first dorsal interosseous muscle. Active EMG electrodes were positioned over the muscle bulk, with signals referenced to a passive electrode placed on the distal radial bone. The EMG signals were recorded at 1 kHz with a passband of 10–330 Hz, time-locked to the MEG data, using a bipolar channel of the MEG system.

### 2.4. MEG Preprocessing

Continuous MEG data were preprocessed offline using MaxFilter 2.2.10 (Elekta Oy, Helsinki, Finland), which included head movement compensation. Temporal signal-space separation (tSSS) preprocessing was applied with a correlation limit of 0.9 and a segment length matching the recording duration. Raw data files were imported into MNE Matlab, and unless otherwise specified, all further analyses were performed using custom Matlab scripts (MathWorks, Natick, MA, USA). Independent component analysis (ICA) was applied to MEG signals filtered between 1 and 25 Hz using the FastICA algorithm (dimension reduction: 30; nonlinearity: tanh) [31]. Between one and three components corresponding to eye blinks and heartbeat artifacts were visually identified based on their topography and time series. These identified components were then removed from the full-band, full-rank MEG signals.

Further analyses were conducted on the data from the MEG sensor that best picks up the right sensorimotor cortex beta rhythm (MEG_SM1_). More precisely, MEG_SM1_ was the MEG signal coming from the sensor showing the highest level of CMC in the beta band, as performed in [4]. The clean MEG_SM1_ and EMG time series were band-pass filtered in the beta frequency range (13–30 Hz), yielding the β_MEG_SM1_ and β_EMG signals. After that, the signals were segmented into 10 s time windows. Afterward, LE, FD, and CD were estimated for β_MEG_SM1_ and β_EMG in each time window.

### 2.5. Lyapunov Exponent

LE assesses the sensitivity of a dynamical system to initial conditions. It estimates how quickly the trajectories of two initially neighboring points converge or diverge over time. A positive LE signifies that trajectories originating from nearby initial states diverge exponentially over time, reflecting chaotic behavior. We chose Rosenstein algorithm due to its proven track record in the accuracy and reliability of feature extraction in the case of chaos estimation of numerous biophysical signals and systems [32]. The steps of the modified Rosenstein algorithm for the estimation of LLE can be found in detail in [32]. The estimation of LE requires a reconstruction of the signal state space, which was created using time delay and embedding dimension, which were calculated by Mutual Information (MI) and False Nearest Neighbors (FNN), respectively [12].

### 2.6. Fractal Dimension

FD quantifies the structural complexity of a dynamical system by assessing how self-similarity persists across different scales. A higher FD indicates a more complex, less predictable signal and a higher degree of self-similarity, whereas a lower FD suggests greater regularity and self-organization. The Higuchi method was chosen since it is particularly valued for its efficiency and robustness in handling various types of time series data [32]. Higuchi’s method of calculation is described in detail in [32].

### 2.7. Correlation Dimension

CD is a mathematical measure used to quantify the complexity and structure of a dynamic system by analyzing how points in a system’s state space correlate with each other at different scales. A higher CD indicates a more intricate, less predictable system, while a lower CD suggests greater regularity and organization. It is a key parameter in nonlinear dynamics and chaos theory, often used to assess the dimensionality of an attractor in a system. CD was estimated using the Grassberger–Procaccia algorithm [9,33].

### 2.8. Burstiness

Beta-band burstiness was estimated using the coefficient of variation (CV) of the beta-band amplitude envelope, with higher CV values reflecting increased presence of transient signal peaks, typically associated with beta bursts. In brief, the β_MEG_SM1_ and β_EMG signals were first filtered between 13 and 30 Hz using a zero-phase FFT-based cosine filter. The analytic envelope was extracted using the Hilbert transform. To correct for slow fluctuations, the envelope was divided by a low-pass filtered version of itself (cutoff = 0.1 Hz), isolating relative amplitude fluctuations. Burstiness was then quantified as the coefficient of variation of this normalized envelope, calculated for each subject.

### 2.9. Statistical Analysis

To investigate differences in nonlinear dynamics between β_MEG_SM1_ and β_EMG, we compared LE, FD, and CD using paired-sample *t*-tests. These comparisons were conducted across subjects to evaluate whether the mean values of each measure significantly differed between the two signal types. Statistical significance was set at *p* < 0.05.

To assess the linear relationship between β_MEG_SM1_ and β_EMG, we computed Pearson’s correlation coefficient of these signals’ envelope. Each envelope was extracted using the Hilbert transform and was further normalized by its low-pass filtered version at 0.1 Hz. Finally, the correlation coefficient was computed between the normalized amplitude envelopes of β_MEG_SM1_ and β_EMG. The resulting subject-wise correlation coefficients were then tested against zero using a one-sample *t*-test to determine whether the group-level mean correlation was significantly different from zero. Moreover, this was performed separately for each of the three nonlinear features (LE, FD, and CD) between β_MEG_SM1_ and β_EMG. Finally, a post hoc power analysis was conducted on the main comparisons (LE, FD, CD) to evaluate the statistical power and robustness of the findings. All analyses were performed using MATLAB 2024b.

To determine the link between classical and nonlinear features of beta oscillations, Pearson correlation was assessed across subjects between burstiness and each of the 3 nonlinear features.

### 2.10. Surrogate Signal Analysis for Nonlinearity Validation

To determine whether nonlinear dynamic features (LE, FD, and CD) reflected structured dynamics rather than noise or spectral properties alone, phase-randomized surrogate signals were generated for each β_MEG_SM1_ and β_EMG recording [34]. These surrogates preserved the original signal’s amplitude spectrum but randomized the phase, thereby disrupting nonlinear temporal dependencies while maintaining spectral characteristics.

For each original signal, 100 surrogate realizations were generated and normalized to match the preprocessing of the real data. Nonlinear features were then computed for each surrogate signal using the same analysis pipeline and parameters. For statistical comparison, a single value per feature was obtained for each subject as the mean of the corresponding 100 surrogate features. Original and surrogate values were compared across subjects using paired *t*-tests.

## 3. Results

### 3.1. Surrogate Analysis Confirms the Validity of Nonlinear Features

Table 1 presents the results of the statistical analysis where original nonlinear features were compared to surrogate ones. Surrogate analysis confirmed the presence of structured, non-random dynamics for two nonlinear features. In fact, both LE and CD showed significantly higher values in the original signals compared to their surrogate counterparts. However, FD failed to reach statistical significance in both β_MEG_SM1_ and β_EMG. The non-significant *p*-values suggest that FD may not effectively distinguish between biologically structured dynamics and spectrally matched noise.

### 3.2. Burstiness Correlates with Nonlinear Features of Beta Activity

Pearson correlation was assessed between the burstiness of the beta envelope, quantified by the CV and three nonlinear dynamic measures (LE, FD, and CD).

Significant correlations were observed for both LE and CD in β_MEG_SM1_, with LE negatively correlating with burstiness (r = –0.60, *p* = 0.009), and CD showing a very strong positive association (r = 0.97, *p* < 0.001). These results suggest that increased beta burstiness is associated with more pronounced nonlinear dynamics in β_MEG_SM1_ signals. In contrast, FD did not show a significant correlation (r = 0.16, *p* = 0.51).

For β_EMG, CD also correlated significantly with burstiness (r = 0.56, *p* = 0.01), while LE and FD showed no reliable relationship (*p* = 0.81 and *p* = 0.20, respectively).

### 3.3. Lyapunov Exponent

**Figure 2:**
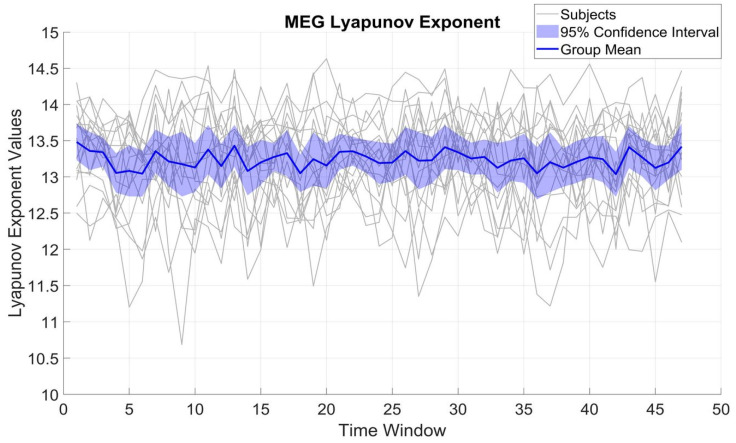
Temporal evolution of the LE in β_MEG_SM1_ activity across all 10 s time windows. Gray lines represent individual subject trajectories (N = 17), while the thicker blue line shows the group’s mean. The shaded area denotes the 95% confidence interval on group mean values.

Figure 2 shows the variation of LE over different time windows for β_MEG_SM1_. LE variations were within a rather limited range of 2 to 3 units for each subject, with a standard deviation (SD) of 0.64 and a mean of 13.22. The limited SD compared to the mean implies that β_MEG_SM1_ for each subject preserves a relatively constant complexity over time.

**Figure 3:**
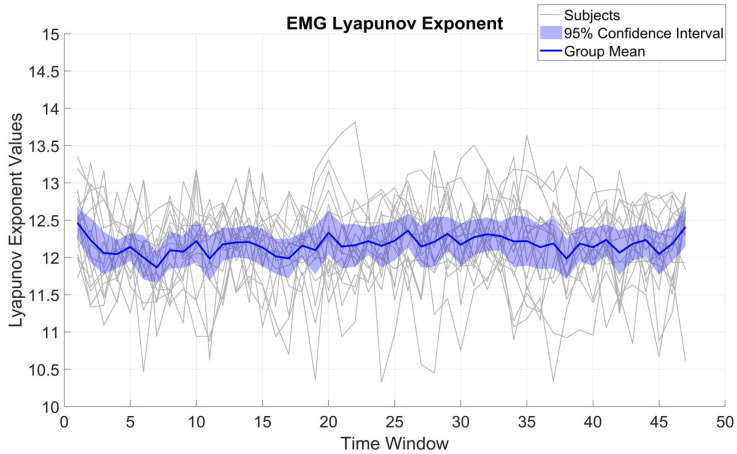
Fluctuations of the LE in β-band EMG activity across all 10 s time windows for all subjects. All is as in Figure 2.

Figure 3 displays LE variations for β_EMG. Across subjects, LE displayed an SD of 0.54 and a mean of 12.17. Compared to β_MEG_SM1_, the β_EMG values were significantly smaller (t(16) = 8.16, *p* < 001). The SD of the β_EMG LE values was not significantly different from that of β_MEG_SM1_ (t(16) = 0.44, *p* = 0.66), implying β_EMG is also quite stable over time and indicates that the overall β_EMG system complexity does not change appreciably over the course of the task.

### 3.4. Correlation Dimension

**Figure 4:**
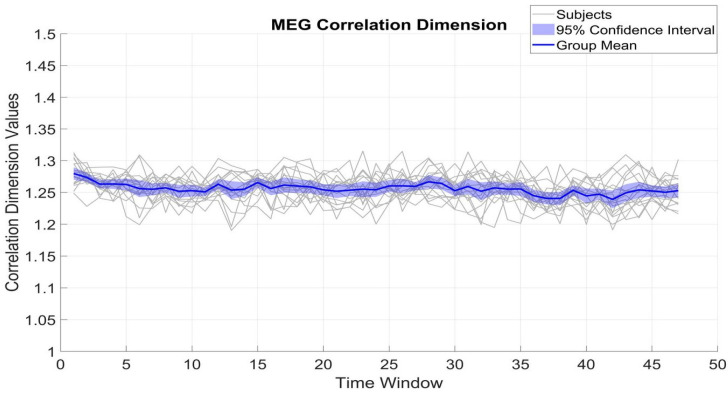
Temporal evolution of the CD in β_MEG_SM1_ activity. All is as in Figure 2.

**Figure 5:**
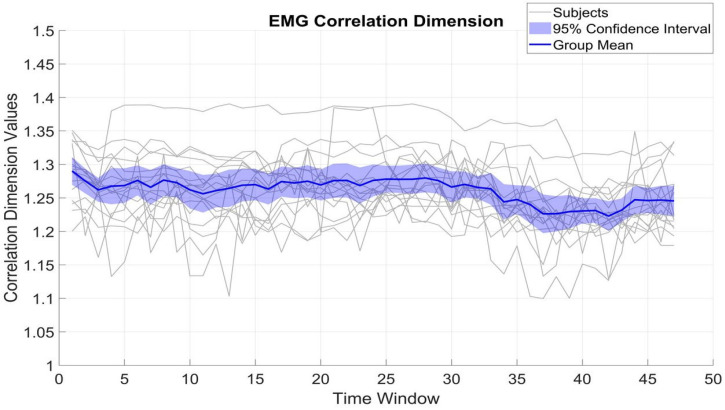
Fluctuations of the CD in β-band EMG activity. All is as in Figure 2.

Figure 4 and Figure 5 show the fluctuation of CD of β_MEG_SM1_ and β_EMG across different time windows, which serve to measure the dimensional complexity and stability of neural and muscular activity. The CD values of β_MEG_SM1_ show a tendency to be sustained and are consistently between 1.2 and 1.35. The small standard deviation (0.02) from the mean (1.25) among subjects indicates low inter-subject variability.

In contrast, β_EMG shows a greater degree of variability, with the CD values ranging between 1.1 and 1.4, with an SD of 0.05. Despite this broader range and variability, the mean CD of β_EMG remains relatively stable at around 1.26. The differences between β_MEG_SM1_ and β_EMG (t(16) = 1.63, *p* = 0.12) reflect underlying variations in the generation and regulation of neural versus muscular signals.

Table 2 presents the mean ± standard deviation of the nonlinear features (LE, FD, CD) for β_MEG_SM1_ and β_EMG, along with the results of the paired-sample *t*-tests.

### 3.5. Comparing MEG and EMG Similarity: Linear (Correlation and Coherence) vs. Nonlinear Features

We used Pearson’s correlation coefficient to quantify the strength of the linear relationship between the envelope of β_MEG_SM1_ and β_EMG. The correlation coefficient between these signals was 0.15 ± 0.09, which was significantly positive (t(16) = 6.63, *p* < 0.001). Therefore, this linear feature could capture some degree of similarity between β_MEG_SM1_ and β_EMG. In contrast, although both MEG and EMG signals demonstrated significant, stable nonlinear dynamics during the task, as shown by nonzero values, there was no significant relationship between β_MEG_SM1_ and β_EMG in the three explored nonlinear features (Table 3).

### 3.6. Post Hoc Power Analysis

Post hoc power analyses were conducted for the paired-sample t-tests comparing the nonlinear measures between β_MEG_SM1_ and β_EMG. For the LE, the effect size (Cohen’s d) was 1.98, yielding a statistical power of 0.99, indicating a very high likelihood of detecting a true effect. FD exhibited an effect size of 4.22 with a corresponding power of 1.00. In contrast, CD had a smaller effect size of 0.39 and a power of 0.32, suggesting that the test was underpowered for this measure. These results support the robustness of the observed significant differences in LE and FD, while the nonsignificant findings for CD may reflect insufficient power rather than a true absence of effect.

## 4. Discussion

In the current study, nonlinear dynamic features including LE, FD, and CD were used to assess how beta oscillations seen in MEG (β_MEG_SM1_) and EMG (β_EMG) signals behave over time and relate to each other during an isometric task. The use of nonlinear analysis is justified by the transient, burst-like nature of beta oscillations, observable in both MEG and EMG, which is a hallmark of nonlinear dynamics [35].

We found that neural (β_MEG_SM1_) and muscular (β_EMG) signals displayed similar nonlinear dynamics, characterized by temporal stability. However, these features were not significantly correlated between β_MEG_SM1_ and β_EMG, in contrast with more classical features obtained with correlation-based analysis.

CD showed a strong relationship with beta burstiness, particularly in β_MEG_SM1_, which suggests that it reflects similar information to that captured by the amplitude envelope. In comparison, LE appeared to capture other aspects of signal complexity that were not directly related to burstiness. FD, on the other hand, did not differ from surrogate data and showed no clear link to burstiness, indicating limited sensitivity to meaningful physiological patterns in this task.

### 4.1. Validity of Nonlinear Metrics

One of the primary goals of this study was to assess the biological validity of nonlinear signal properties using surrogate data. Both LE and CD significantly distinguished real signals from phase-randomized surrogates, supporting their sensitivity to structured, nonlinear dynamics. In contrast, FD failed to meet conventional significance thresholds, undermining its robustness in this context. Additionally, FD showed no significant correlation with beta burstiness, further questioning its relevance for capturing biologically meaningful variability during isometric contraction. By comparison, CD demonstrated a very strong correlation with β_MEG_SM1_ and a moderate one with β_EMG, suggesting it tracks beta burst dynamics closely, potentially even reflecting properties already captured by the variability of the amplitude envelope. Meanwhile, LE exhibited either inverse or nonsignificant associations with burstiness, indicating it may reflect orthogonal aspects of complexity not captured by envelope variability. This divergence implies that while CD aligns with classical envelope-based properties of the signal, LE may provide complementary, less redundant, insights into the intrinsic dynamical organization of cortical and muscular systems.

### 4.2. Neural and Muscular Stability Reflects Functional Demands

Both beta band MEG (β_MEG_SM1_) and EMG (β_EMG) signals demonstrated a high degree of temporal stability for their nonlinear properties despite the relatively small fluctuation within subjects, suggesting that both neural and muscular systems maintain consistent dynamical behavior under the conditions of an isometric task. This consistency is a functional necessity, as effective motor output during steady isometric tasks relies on minimizing fluctuations in both neural drive and muscular execution [36]. In other words, motor systems use a stable control strategy to ensure precise sustained muscle force generation.

### 4.3. Cross-Modal Relationship and Methodological Implications

The correlation analysis showed a small but significant positive relationship between β_MEG_SM1_ and β_EMG in terms of envelope coupling. It means that their modulation is similar in the beta band during isometric contractions. This finding is consistent with previous works that used coherence-based analysis and supports the idea that EMG may serve as a proxy of central neural activity [27,28]. However, nonlinear features (LE, FD, CD) did not show significant cross-modal correlations. This may be because these nonlinear metrics depend on assumptions of deterministic and low-noise dynamics, which are often not met in real-world neurophysiological recordings. Moreover, nonlinear metrics are known to be highly sensitive to preprocessing decisions, segmentation strategies, and noise contamination, which could hide true relationships between cortical and muscular nonlinear metrics [9,35]. Therefore, while the absence of cross-modal similarity in nonlinear metrics might reflect distinct underlying mechanisms, alternative explanations linked to the limited signal-to-noise ratio cannot be ruled out.

This large difference raises a crucial methodological issue: linear measures, such as coherence or correlation, yield more reliable measures of similarity in noisy biosignals. Nonlinear measures are more sensitive to internal dynamic complexity and structural features of the signal’s trajectory in reconstructed state-space but are less robust for inter-system comparisons unless recording conditions are highly controlled.

### 4.4. Comparative Dynamics of Brain and Muscle Systems

The greater LE of β_MEG_SM1_ compared to β_EMG indicates fundamental differences of cortical versus muscular systems in terms of dynamic properties. LE quantifies the sensitivity of a system to initial conditions, referring to how two nearly different trajectories diverge over time in phase space [9]. In neural concept, it means that small perturbations like sensory input or cognitive changes can quickly lead to different (divergent) patterns of cortical activity. To clear it more, if we have one cortical state representing activity in the absence of a stimulus and another state begins with a minimal stimulus, we will obtain different neural patterns and LE values. This flexibility is vital for integrating sensory feedback, error correction, and cognitive input [37]. In contrast, the lower LE in β_EMG suggests that muscular dynamics are less responsive to small changes and are governed by more stable, deterministic patterns, such as spinal level movement pattern generators, i.e., the lower motoneuron circuits in the spinal cord. In addition, the limited flexibility of EMG may also reflect physiological constraints inherent to the motor units themselves. These units have relatively narrow firing rate ranges (typically from 0 to ~30 Hz) [38], unlike cortical neurons that can operate across a much broader frequency spectrum [39]. As a result, the interference EMG signal, generated from the summation of motor unit action potentials, is inherently less dynamic. This indicates that muscles play as a consistent executor of motor commands prioritizing reliable and repeatable output over dynamic flexibility. This is aligned with hierarchical predictive coding models, where higher-order brain areas plan and adapt motor behavior, while peripheral systems such as muscles are specialized for precise, stable execution [40,41].

### 4.5. Practical Implications for Motor Control and Applied Contexts

The observed stability of nonlinear dynamics in both cortical and muscular beta activities may have meaningful implications for applied fields such as motor rehabilitation and high-performance sports. The ability of the motor system to maintain stable complexity under steady conditions suggests an optimized balance between flexibility and robustness, a property that may be disrupted in neurological disorders like Parkinson’s disease [42]. So, quantifying this stability using nonlinear features like LE or CD may serve as a potential biomarker for motor system health or adaptability. In addition, in sports science, athletes sometimes need to perform tasks requiring sustained isometric contractions or precise motor control under pressure, like planche in gymnastics. Understanding how neural and muscular systems maintain or lose stability under load could help tailor training programs aimed at optimizing neuromuscular efficiency and preventing injury. Similarly, in clinical rehabilitation, deviations from normative nonlinear dynamics might help identify patients who are at risk of poor motor recovery or who may benefit from neuromodulatory interventions like brain stimulation or biofeedback [43]. Therefore, while this study is exploratory in scope, it paves the way for future studies aiming to assess the applicability of nonlinear metrics of brain and muscle beta oscillations for diagnostic or performance assessments.

### 4.6. Limitations and Future Directions

There are some limitations in this study that need to be considered. Firstly, the experimental design focused exclusively on a steady isometric contraction task involving a single hand. While this controlled setup helps reduce variability and isolate beta-band dynamics, it inherently limits the ecological validity and broader applicability of the findings. Future studies should assess how complexity in different tasks such as dynamic contractions and multi-joint coordination affects nonlinear dynamics. In addition to this, the generalizability of these findings to more naturalistic or complex motor behaviors, or to clinical populations with altered neuromuscular control, remains uncertain. Therefore, future work is necessary to determine whether the observed patterns hold under a wider variety of movement conditions and subject populations.

Another limitation is the relatively small sample size (17 subjects), which may limit the statistical power of certain comparisons, particularly in the case of CD, where the difference between β_MEG_SM1_ and β_EMG was not statistically significant. Future studies with larger sample sizes are needed to better resolve these subtle dynamics.

Moreover, the assumptions underpinning nonlinear dynamic metrics may not fully apply to hybrid biological systems where stochastic and deterministic processes coexist. LE, FD, and CD, while powerful, are sensitive to signal noise and the choice of embedding parameters, potentially limiting their interpretability under uncontrolled conditions.

Although we observed stable nonlinear features (LE, FD, CD) in both MEG and EMG during isometric contraction, the interpretation of this stability remains uncertain. Future studies should determine whether the observed signal stability reflects meaningful physiological organization or instead arises from methodological constraints such as averaging or limited metric sensitivity.

A promising direction for future research is to apply this same framework to patients with motor disorders during isometric tasks, where potential deviations from healthy dynamic patterns could reveal early biomarkers of motor dysfunction. In addition, investigating how these nonlinear features respond to interventions such as medication or deep brain stimulation could provide insight into their potential as markers of therapeutic efficacy.

The results indicate that linear methods including correlation coefficient and coherence analysis provide more stable measures of similarity between β_MEG_SM1_ and β_EMG because of their lower sensitivity to noise variability induced by pre-processing.

Our analysis selected the MEG sensor based on the highest CMC in the beta band, a linear metric. While this approach ensures functional relevance to motor control, it introduces a methodological limitation by potentially biasing the analysis in favor of linear similarity. This may partly explain why a linear correlation between MEG and EMG beta envelope was significant while no association was observed in nonlinear comparisons. To address this limitation, future studies could consider alternative sensor selection strategies to ensure a more neutral basis for cross-modal comparisons.

Future work could present the topographic maps of the coefficient of variation of the envelope and of nonlinear features, averaged across subjects. In this way it would be possible to compare the modulation of them particularly for assessing enhancement or suppression above sensorimotor regions. This could also help clarify the functional relevance of these features in relation to motor cortical areas.

Finally, nonlinear features used in this study primarily assume deterministic dynamics, which may not fully capture the hybrid stochastic-deterministic nature of real neurophysiological systems [5]. Future studies could integrate hybrid models or stochastic nonlinear measures that may offer a more complete characterization of cortical and muscular electrophysiological complexity.

## 5. Conclusions

This study demonstrates that in healthy individuals, nonlinear dynamic features including LE and CD maintain stability in the beta range over time. This finding validates the approaches based on taking averages in time series. Notwithstanding, nonlinear features could not capture the existent similarity proved by classical methods including envelope correlation and burstiness assessed with CV. This discrepancy suggests that while nonlinear features capture the intrinsic complexity of each signal, they may not be sensitive to the functional connectivity or shared dynamics between brain and muscle activity.

## Figures and Tables

**Table 1:** Statistical comparison of nonlinear dynamic measures between original and surrogate signals for β_MEG_SM1_ and β_EMG.

Metric	Signal Type	t(16)(1)	*p*-Value
LE	β_MEG_SM1_	14.8	<0.001
LE	β_EMG	4.89	<0.001
FD	β_MEG_SM1_	1.83	0.08
FD	β_EMG	1.98	0.06
CD	β_MEG_SM1_	5.75	<0.001
CD	β_EMG	2.34	0.03

**Table 2:** The differences in nonlinear dynamics between β_MEG_SM1_ and β_EMG.

Feature	β_MEG_SM1_ (Mean ± SD)	β_EMG (Mean ± SD)	t(16)	*p*-Value
LE	13.22 ± 0.64	12.17 ± 0.54	8.16	<0.001
FD	1.03 ± 0.001	1.04 ± 0.002	17.4	<0.001
CD	1.25 ± 0.02	1.26 ± 0.05	1.63	0.12

**Table 3:** Pearson correlation coefficients between the amplitude envelopes of β_MEG_SM1_ and β_EMG, and between each of the 3 nonlinear dynamic features of the two signals.

Feature Compared	Correlation Coefficients (Mean ± SD)
Amplitude Envelope	0.15 ± 0.09
LE	−0.004 ± 0.07
FD	0.031 ± 0.028
CD	0.08 ± 0.033

## Data Availability

The data presented in this study are available on request from the corresponding authors because the data are not publicly available due to privacy or ethical restrictions. The Matlab analysis code underlying the reported results is available at http://github.com/ArminH69/Nonlinear-Dynamics-of-MEG-and-EMG-Stability-and-Similar-ity-Analysis accessed on 12 June 2025.

## Abbreviations

The following abbreviations are used in this manuscript:

MEG	Magnetoencephalography
EMG	Electromyography
LE	Lyapunov Exponents
FD	Fractal Dimension
CD	Correlation Dimension
CV	Coefficient of Variation
